# Beta-blockers and Short-Term Cardiovascular Outcomes In Patients Hospitalized For Acute Coronary Syndrome and a Left Ventricular Ejection Fraction ≥40%

**DOI:** 10.1038/s41598-020-60528-y

**Published:** 2020-02-26

**Authors:** Charbel Abi Khalil, Mohammad Zubaid, Menatalla Mekhaimar, Nidal Asaad, Ziyad Mahfoud, Jassim Al Suwaidi

**Affiliations:** 1 Research Department, Weill Cornell Medicine-Qatar, Doha, Qatar; 2 Joan and Sanford I. Weill Department of Medicine, Weill Cornell Medicine, New York, USA; 3 Adult Cardiology, Heart Hospital- Hamad Medical Corporation, Doha, Qatar; 4 Department of Medicine, Faculty of Medicine, Kuwait University, Kuwait, Kuwait; 5 Department of Medical Education, Weill Cornell Medicine-Qatar, Doha, Qatar; 6 Department of Healthcare Policy and Research, Weill Cornell Medicine, New York, USA

**Keywords:** Cardiology, Medical research

## Abstract

Beta-blockers (BB) have been traditionally associated with improvement in cardiovascular disease outcomes in patients with ischemic cardiomyopathy. Whether they’re still efficacious in the post-reperfusion era is currently debated in the light of recent controversial reports. In-hospital, 6-month and 12-month mortality were studied in the GULF-COAST, a prospective multicenter cohort of acute coronary syndrome (ACS), in relation to BB use: prior to admission, 24-hour post-admission and on discharge in patients with a left ventricular ejection fraction (LVEF) ≥ 40%. On admission, 50.9% of the cohort participants had a LVEF ≥ 40%, of whom 1203 (55.4%) were on BB whilst 905 (44.6%) were not. Mean age was 60 (13) years old and 66% were males. Prior BB use or its administration in 24 hours decreased in-hospital mortality (OR = 0.25, 95% CI [0.09–0.67]; OR = 0.16, 95% CI [0.08–0.35]; respectively). BB on discharge lowered 1-month mortality (OR = 0.28, 95% CI [0.11–0.72]), but had a neutral effect on mortality, reinfarction and stroke at 6 and 12 months. Results were unchanged after multivariable adjustments and further sensitivity analysis. In this retrospective cohort of ACS, BB improved in-hospital and 1-month mortality in patients with a LVEF ≥ 40% but had a neutral effect on longer-term outcome.

## Introduction

Several trials conducted in the late 1970’s and 1980’s, such as ISIS-1 (First International Study of Infarct Survival)^1^ and BHAT (Beta-Blocker Heart Attack Trial)^2^, showed that beta blockers (BB) decrease mortality after myocardial infarction (MI). An earlier meta-analysis of studies in which MI patients were treated with BB reported a 25 percent reduction in one-year mortality^3^.

The treatment of ischemic cardiomyopathy has been revolutionized during the past 2 decades with the introduction of new treatment regimens such as dual anti-platelets, statins and most importantly reperfusion therapy. Progressively, the long-term protective role of BB in MI, once vital, is being questioned. A large metanalysis that included over 100 000 MI patients showed that BB reduce mortality in the pre-reperfusion era but failed to report any long-term survival benefit of BB in trials performed in the post-reperfusion era^4^.

There is still convincing evidence that BB use is beneficial on the short-term outcome. A 2013 meta-analysis of randomized trials concluded that early BB therapy in ACS patients reduces in-hospital mortality, re-infarction and arrhythmias^5^. Nevertheless, it is not known how long BB treatment beneficial post-ACS is. In this paper, we report that previous BB therapy and/or BB treatment up to 24 hours after admission is associated with improved in-hospital outcome. However, BB therapy on discharge was associated only with decreased 1-month mortality, with no effect on mortality at 6 and 12 months.

## Results

### In-hospital outcome

**Figure 1:**
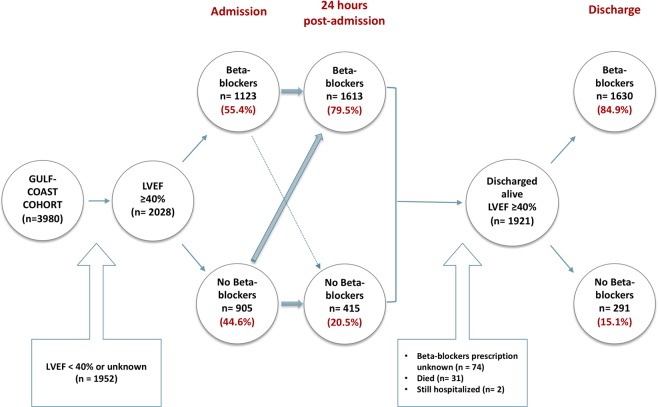
Flow chart of the study analysis.

A total of 3980 patients participated in the Gulf COAST cohort. Out of those, 2028 fulfilled the inclusion criteria (LVEF ≥ 40%) and were included in the analysis (see flow chart- Fig. 1). Among those, 1123 (55.4%) of patients were on a BB on admission whilst 905 (44.6%) were not. Mean age was 60(13) and 66% were males. Table 1 shows the baseline characteristics of the study groups according to BB on admission. Although they were similar in gender and age, patients on BB had more comorbidities. They had a higher prevalence of dyslipidemia, hypertension and MI, which could probably explain the higher prescription of cardioprotective medications such as aspirin, statins and ACE-inhibitors/ARBs. As expected, the heart rate was lower under BB treatment (82.9 vs 86.9 bpm, p < 0.001).

**Table 1:** Baseline characteristics of patients admitted for ACS at the GULF-COAST, with a LVEF > = 40%, according to beta-blockers on admission.

Variable	Beta-blockers on admission N = 1123 (55.4%)	No beta-blockers on admission N = 905 (44.5%)	P value
**Demographics**			
Age (years)	62.3 ± 11.6	61.2 ± 11.9	0.08
Gender (Male)	470 (59.6%)	376 (59.3%)	0.92
BMI (kg/m^2^)	29.7 ± 9.5	29.7 ± 6.5	0.94
Smoking (current)	137 (17.4%)	142 (22.4%)	0.017
**Past medical history**			
Dyslipidemia	595 (75.4%)	393 (62.0%)	<0.001
Hypertension	692 (87.7%)	500 (78.9%)	<0.001
Diabetes	491 (62.2%)	396 (62.5%)	0.92
Myocardial infarction	290 (36.8%)	88 (13.9%)	<0.001
Stroke/TIAs	66 (8.4%)	50 (7.9%)	0.46
PAD	25 (3.2%)	15 (2.4%)	0.36
Cancer	9 (1.1%)	9 (1.4%)	0.64
Venous thrombo-embolism	3 (0.4%)	5 (0.8%)	0.47
CKD	79 (10.0%)	37 (5.8%)	0.004
**Clinical parameters**			
SBP, mmHg, mean ± SD	144.2 ± 28.5	145.0 ± 26.4	0.58
DBP, mmHg, mean ± SD	80.0 ± 16.0	82.1 ± 15.7	0.01
HR, bpm, mean ± SD	82.9 ± 20.0	86.9 ± 21.1	<0.001
LVEF, %	53.2 ± 8.3	53.9 ± 8.4	0.11
**Medications prior to admission**			
Aspirin	680 (86.2%)	385 (60.7%)	<0.001
ACE-inhibitors and/or ARBs	566 (71.7%)	424 (66.9%)	0.048
Statins	692 (87.7%)	419 (66.1%)	<0.001
Clopidogrel or prasugrel	286 (36.2%)	79 (12.5%)	<0.001

Data are shown as number (percentage) or mean ± standard deviation. TIAs = transient ischemic attacks, PAD = peripheral artery disease, CKD = chronic kidney disease, SBP = systolic blood pressure, DBP = diastolic blood pressure, LVEF = Left ventricular ejection fraction, HR = heart rate, ARBs = angiotensin receptor blockers.

Within the first 24 hours, 1613 (79.5%) patients were on BB whilst 415 (20.5%) were not. The distribution of cardiometabolic parameters and baseline characteristics in those 2 groups was not significantly different from the one done according to prior BB use at admission.

A total of 31 in-hospital deaths occurred, including one death in a patient who was not on BB on admission and died before receiving any within the first 24 hours. Mortality was lower in patients on BB prior to admission or who received it in 24 hours (OR = 0.25 95% CI [0.09–0.67], *p* = 0.007; OR = 0.16 95% CI [0.08–0.35], *p* < 0.001; respectively).Results were unchanged after further multivariable adjustments (OR = 0.28 95% CI [0.09–0.82], *p* = 0.018; OR = 0.18 95% CI [0.08–0.39], *p* = 0.001; respectively). There was no association between prior BB use or its administration in 24 hours, and in-hospital mortality by the presence of revascularization (p = 0.551; p = 0.381; respectively) or by the type of ACS (p = 0.454; p = 0.842; respectively).

### Long-term outcome

1921 patients with a LVEF ≥ 40% were discharged alive (Fig. 1), the majority of whom were treated with BB (84.9%). As shown in Table 2, there were more males (68% versus 51.9%, *p* < 0.001) in the BB group. Those patients were more likely to have had STEMI (26.7% vs 17.2%, *p* = 0.001) and received more cardioprotective drugs at discharge whereas NSTEMI was more prevalent in the non-BB group. There was a non-significant higher use of thrombolysis and PCI in the BB group. However, the severity of lesions was homogenous among patients who underwent PCI.

**Table 2:** Baseline characteristics of patients discharged alive from ACS in the GULF-COAST, with a LVEF > = 40%, according to beta-blockers on discharge.

Variable	Beta-blockers on discharge N = 1630 (84.9%)	No beta-blockers on discharge N = 291 (15.1%)	p
**Demographics**			
Age (years)	59.3 ± 12.3	60.8 ± 14.0	0.08
Gender (Male)	1109 (68.0%)	151 (51.9%)	<0.001
BMI (kg/m^2^)	29.4 ± 8.0	30.8 ± 9.6	0.21
Smoking (current)	417 (25.6%)	73 (25.1%)	0.85
**Past medical history**			
Dyslipidemia	886 (54.4%)	160 (55.0%)	0.84
Hypertension	1039 (63.7%)	193 (66.3%)	0.39
Diabetes	839 (51.5%)	144 (49.5%)	0.53
Myocardial infarction	317 (19.4%)	50 (17.2%)	0.36
Stroke/TIAs	97 (6.0%)	19 (6.5%)	0.70
PAD	30 (1.8%)	9 (3.1%)	0.16
Cancer	24 (1.5%)	3 (1.0%)	0.78
Venous thrombo-embolism	4 (0.2%)	1 (0.3%)	0.56
CKD	95 (5.8%)	14 (4.8%)	0.49
**Clinical parameters**			
SBP, mmHg, mean ± SD	144.0 ± 26.8	139.2 ± 27.5	0.005
DBP, mmHg, mean ± SD	82.1 ± 15.4	77.5 ± 15.3	<0.001
HR, bpm, mean ± SD	83.9 ± 19.7	81.8 ± 22.1	0.12
LVEF, %	53.2 ± 8.2	52.8 ± 8.4	0.46
**Medications at discharge**			
Aspirin	1588 (97.4%)	269 (92.4%)	<0.001
ACE-inhibitors and/or ARBs	1380 (84.7%)	199 (68.4%)	<0.001
Statins	1603 (98.3%)	268 (92.1%)	<0.001
Clopidogrel or prasugrel	1259 (77.2%)	184 (63.2%)	<0.001
**Discharge diagnosis**			
STEMI	387 (26.7%)	45 (17.2%)	0.001
NSTEMI	659 (45.4%)	136 (52.1%)	0.046
Unstable Angina	405 (27.9%)	80 (30.7%)	0.36
**Treatment**			
PCI*	452 (27.7%)	65(22.3%)	0.056
Thrombolysis	239 (14.7%)	30 (10.3)	0.054
CABG	39 (2.4%)	5 (1.7%)	0.47
Severity of lesions**			
1 artery	73 (34.2%)	74 (46.2%)	0.18
2 arteries	69 (32.4%)	35 (21.9%)	
3 arteries	64 (30%)	35 (21.9%)	
4 arteries	7 (3.3%)	4 (2.5%)	

Data are shown as number (percentage) or mean ± standard deviation. TIAs = transient ischemic attacks, PAD = peripheral artery disease, CKD = chronic kidney disease, SBP = systolic blood pressure, DBP = diastolic blood pressure, HR = heart rate, LVEF = Left ventricular ejection fraction, ARBs = angiotensin receptor blockers, STEMI- ST elevation myocardial infarction, NTSEMI = Non- ST-elevation myocardial infarction. PCI = percutaneous coronary intervention, CABG = coronary artery bypass graft. *Including primary PCI. **Among patients who underwent PCI.

At 1 month, there were 18 deaths, giving a cumulative mortality of 2.4%. The mortality was lower in the BB group (OR 0.28, 95% CI 0.11–0.72, *p *= 0.008), and remained so after multivariable analysis (OR 0.25, 95% CI 0.09–0.67, *p *= 0.006). There was no association between BB on discharge and one-month mortality by the presence of earlier revascularization at the hospital (p = 0.997) or by the type of ACS (p = 0.995).

At 6 and 12 months, the cumulative mortality was 4.8% and 7.2%; respectively. However, the protection conferred by BB was lost. Similarly, there was no significant difference in the 12-month incidence of reinfarction or stroke (Table 3).

**Table 3:** Medium-term outcomes of patients admitted for acute coronary syndrome at the GULF-COAST, with a LVEF > = 40%, according to beta-blockers on discharge.

	1-month mortality	6-month mortality	12-month mortality	12-month re-infarction	12-month stroke
	Number of events (%) OR 95% CI	Number of events (%) OR 95% CI	Number of events (%) OR 95% CI	Number of events (%) OR 95% CI	Number of events (%) OR 95% CI
**Beta-blockers at discharge**					
No	7 (2.5%) OR = 1	8 (3.1%) OR = 1	9 (3.4%) OR = 1	2 (0.7%) OR = 1	2 (0.7%) OR = 1
Yes	11 (0.7%) OR = 0.28 (0.11–0.72)	41 (2.7%) OR = 0.86 (0.40–1.86)	38 (2.5%) OR = 0.74 (0.35–1.55)	17 (1.0%) OR = 1.52 (0.35–6.63)	5 (0.3%) OR = 0.44 (0.09–2.30)
P value	0.008*	0.71	0.42	0.57	0.33

### Sensitivity analysis

511 patients with BB prior to admission were propensity-score matched with other 511 patients with no BB. Both groups were well balanced for baseline characteristics expect for dual anti-platelet therapy (DAP) that was more often prescribed in patients on BB (Supplementary Table 1). Previous BB therapy was associated with lower mortality (OR 0.30, 95% CI 0.11–0.84, *p *= 0.022), which even remained statistically significant after correction for cofounding factors (OR 0.31, 95% CI 0.11–0.87, *p *= 0.027). On discharge, 291 patients with BB were propensity scored to 291 patients without BB. Both groups were also well balanced for anthropometric measures, discharge diagnosis, severity of coronary lesions, but not for the treatment they received. The BB group was more likely to have had a PCI during hospital stay and a DAP on discharge. (Supplementary Table 2). There was a total of only 8 deaths at one month: 1 death in the BB group and 7 in the non-BB group; hence BB conferred protection against mortality although the statistical significance *per se* was lost after correction for parameters that were not balanced in the propensity model (OR 0.17, 95% CI 0.02–1.17, *p* = 0.067). However, 6-month and 12-month mortality were not affected by BB prescription at discharge (OR 0.95, 95% CI 0.35–2.58, *p* = 0.92; OR 0.77, 95% CI 0.28–2.11, *p* = 0.61; respectively); neither re-infarction or stroke (OR 0.95, 95% CI 0.35–2.58, *p* = 0.92; OR 0.77, 95% CI 0.28–2.11, *p* = 0.61; respectively).

## Discussion

We showed in this retrospective cohort of ACS that a previous BB therapy or a BB treatment within 24 hours of admission decreases in-hospital mortality. Further, BB on discharge conferred cardiovascular protection up to one month, but no additional benefit on 6 and 12-month mortality, re-infarction and stroke was observed.

Early reperfusion has become the cornerstone of ACS treatment^6^. Nevertheless, our results support the hypothesis that BB still have a beneficial role in the post-reperfusion era and improve the outcome in the early course of the disease. Interestingly, patients admitted for ACS under BB therapy in our cohort had a significantly lower mortality although they had more comorbidities. It might be possible that BB protect from fatal events despite the occurrence of ischemia. However, we acknowledge that those patients also received more cardioprotective drugs. Further, the BB group had also a higher prescription of cardiometabolic drugs on discharge. Nevertheless, all those confounding factors were accounted for in the multivariable regression model and the sensitivity analysis, which did not abolish the earlier protective role of BB.

There is no doubt that an early BB therapy in ACS is beneficial on the short-term, even in the post -reperfusion era. A recent analysis of the “International Survey of Acute Coronary Syndromes” showed that an early BB administration–defined as an BB intake ≤ 24 hours post-admission- decreases by almost twice in-hospital mortality^7^. Moreover, BB also improved the left ventricular function, a benefit that was also previously reported with early intravenous metoprolol in ST elevation MI patients included in the METOCARD-CNIC trial^8^. In a meta-analysis that included over 70 000 patients, early intravenous BB therapy during ACS reduced mortality, ventricular tachyarrhythmias and reinfarction. In a recent Brazilian observational study, oral BB use within the first 24 hours of ACS onset resulted in decreased in-hospital mortality^9^.

To our knowledge, we are the first to report that BB on discharge from ACS have a short-term benefit that is lost only after 6 months. However, the neutral effect of BB on one-year mortality and beyond has been reported by several observational studies: In the French FAST-MI registry of ACS, BB did not reduce 1-year and 5-year mortality^10^. Similar findings were reported in a sub-study of the British Myocardial Ischemia National Audit Project that assessed one-year mortality in ACS patients with a preserved LVEF^11^. A 2015 meta-analysis of over 40 000 patients admitted for ACS found that the survival benefit of BB is lost beyond 1 year^12^.

It is not known why BB protect only on the short-term. During acute MI, the sympathetic nervous system is activated^13^ and plasma catecholamines are elevated^14^, which increases the risk of arrhythmogenesis^15^. It is believed that BB counteract those deleterious pathophysiological effects, decrease the myocardial oxygen demand and reduce the infarct size^8^; hence decreasing the risk of short-term mortality post-MI. In the current era, early reperfusion, dual anti-platelet therapy, statins use and other treatment have greatly contributed to the preservation of the myocardium and limited its damage post-ACS^16,17^. It might be possible that chronic BB therapy, once recommended to prevent remodeling of the infarct zone in the pre-perfusion era, is not necessary anymore. In a recent study that assessed LV remodeling by repeating echocardiographic measurements 8 months post-MI, BB did not change any LV parameters in patients who had coronary revascularization and received secondary prevention medications^18^.

Beta-blocker therapy has also been the cornerstone treatment of heart failure (HF) for the past 3 decades. Robust data from randomized controlled trials (RCTs) and metanalysis support the use of BB in patients with heart failure and reduced ejection fraction (HFrEF) in chronic, and even in acute HF^19–21^. We have alrerady reported that a previous BB therapy in patients hospitalized for acute HF is associated with decreased in-hospital mortality^22^. Additionally, we have shown that non-withdrawal of BB during acute decompensation is safe and is associated with better in-hospital outcome^23^. It is believed that the protective effect of BB in HFrEF is secondary to their ability to reduce the deleterious effect of chronic β-receptor stimulation (arrhythmias, cardiomyocytes apoptosis and hypertrophy)^24^. However, it is not clear whether any cardiovascular protection could be achieved using beta-blockers in heart failure with preserved ejection fraction (HFpEF) in the presence of 2 inconclusive meta-analysis with a low quality of evidence^21,25^. We have recently shown that a previous beta-blocker therapy had a neutral effect on in-hospital outcome in acute decompensated HFpEF patients with coronary artery disease, a similar outcome at one year was observed with the administration of beta-blockers at discharge from the hospital^26^.

We acknowledge the presence of several limitations in our study. This was not a randomized controlled trial, rather an observational cohort of ACS. Important factors such as the type of BB, its dose and duration in patients already treated on BB on admission, were not recorded. Moreover, patients were not monitored for adherence after discharge, which could have influenced the long-term mortality. In our regression model and in our sensitivity analysis, we included several predictors of mortality. However, we cannot exclude the presence of other factors not recorded in our cohort that could have influenced the outcome. Finally, nearly two-thirds of the patients were not revascularized during their hospital stay; hence, these conclusions may only be relevant to non-revascularized ACS patients.

In summary, this study shows that BB therapy has a beneficial effect on mortality in ACS patients, with a LVEF ≥ 40% when used prior to, or within 24 hours of admission. BB therapy also improves mortality when given on discharge for up to one month. However, no further protective effect on mortality, reinfarction and stroke is observed beyond 6 months. Further studies are needed to clarify whether beta-blocker therapy post-ACS should not be given according to the left ventricular ejection fraction, but rather according to the remaining ischemia burden.

## Methods

### Study group

The Gulf COAST registry is a prospective, multicenter study of ACS patients recruited for 12 months (January 2012 to January 2013), from 4 Middle Eastern Gulf countries: Bahrain, Kuwait, Oman and United Arab Emirates. The study describes clinical characteristics and cardiovascular outcome of patients admitted with ACS. Details pertaining to the study design, methodology and recruitment have been previously published^27^. Briefly, we consecutively enrolled Gulf Nationals from 29 hospitals, 18 years or older, admitted for ACS. Patients were then followed at 1, 6 and 12 months after discharge, at the clinic or by a telephone interview. All cardiovascular outcomes and clinical measurements were defined according to the American College of Cardiology (ACC)/American heart association (AHA) task force on clinical data standards^28^. ACS included unstable angina (UA), non-ST segment myocardial infarction (NSTEMI) and ST segment myocardial infarction (STEMI). The study was approved and oversight by the institutional ethics committee of Kuwait University (Number XX02/11), and subsequently by the local institutional ethics committees of each of the 29 participating hospitals^27^. A written informed consent was obtained from every study participant. The study conformed to the 1975 Helsinki declaration and the STROBE epidemiological reporting guidelines^29^.

In this study, we first assessed in-hospital mortality in ACS patients with a left ventricular ejection fraction (LVEF) ≥ 40%, in relation to BB on admission and at 24 hours post-admission. We than studied mortality in the same group of patients at 1-, 6- and 12-months in relation to BB on discharge. Additionally, reinfarction and stroke were assessed at 12 months.

### Statistical analysis

Baseline categorical variables and outcome measures were summarized using frequency distributions while means and standard deviations were used for continuous variables. Outcome measures and baseline patients’ characteristics were compared between the two groups: BB *versus* no BB using the χ^2^ test (or Fisher’s exact test when expected cell counts fell below 5) for categorical variables and the student’s t test or Wilcoxon rank sum test for numeric variables as previously described^30^. Multivariable logistic regression analysis was performed for mortality when the latter was statistically different in the study groups. The model included variables that were statistically significant between both groups, except for variables that have a high risk of co-linearity, in addition to age, gender. The model for in-hospital mortality included age, gender, smoking, dyslipidemia, hypertension, previous MI, heart rate, aspirin and diabetes. The model for one-month mortality included age, gender, systolic blood pressure (SBP), medications at discharge and discharge diagnosis. In order to test if the association between BB and mortality is different for those with vascularization vs. those without and for those with different types of ACS, interaction terms were included in the logistic regressions. Adjusted Odds Ratios (OR) are presented with their 95% CI and corresponding p values. Statistical significance was set at the 5% level (two-tailed test). All analyses were done using IBM-SPSS version 22.0.

### Sensitivity analysis

We performed a propensity score analysis of participants on BB versus non-BB, on admission and on discharge. Propensity scores were computed using logistic regression with membership in the two groups on 6 baseline variables that are significantly different between the two study arms on admission: age, gender, smoking, dyslipidemia, hypertension, MI (prior to admission) and heart rate, using the 1:1 nearest neighbor matching method with a tolerance level of 0.01. Further multivariable logistic regression analysis was performed and included variables that were still significantly different after propensity matching: Aspirin, ACE-inhibitors/ARBs, statins and clopidogrel/prasugrel for in-hospital mortality in both models.

## Supplementary information

Supplementary information.

## Data Availability

The data that support the findings of this study are available from the authors upon reasonable request and with permission of the GULF-COAST principal investigator.
