# Supplementary material for: Beta-blockers and Short-Term Cardiovascular Outcomes In Patients Hospitalized For Acute Coronary Syndrome and a Left Ventricular Ejection Fraction ≥40%
Source: Sci Rep. 2020 Feb 26;10:3520. doi: 10.1038/s41598-020-60528-y (PMC7044295; doi:10.1038/s41598-020-60528-y)
Supplement: Supplementary file 1 — Supplementary information. [file 41598_2020_60528_MOESM1_ESM.pdf]

Supplementary Table 1: Baseline characteristics of propensity-score matched patients admitted for ACS at the GULF-COAST, with a LVEF >= 40%, according to beta-blockers on admission.

**Variable**	**Beta-blockers on admission**<br><br>**N= 511**	**No beta-blockers on admission**<br><br>**N= 511**	**p**
**Demographics**			
Age (years)	61.6±12.1	61.9±11.7	0.71
Gender (Male)	293 (57.3)	292 (57.1%)	0.95
BMI (kg/m2)	29.6±6.4	30.0±6.7	0.32
Smoking (current)	96 (18.8%)	101 (19.8%)	0.69
**Past medical history**			
Dyslipidemia	349 (68.3%)	350 (68.5%)	0.94
Hypertension	433 (84.7%)	438 (85.7%)	0.65
Diabetes	306 (59.9%)	323 (63.2%)	0.24
Myocardial infarction	90 (17.6%)	87 (17%)	0.80
Stroke/TIAs	36 (7.0%)	44 (8.6%)	0.35
PAD	16 (3.1%)	13 (2.5%)	0.57
Cancer	4 (0.8%)	6 (1.2%)	0.52
Venous thrombo-embolism	2 (0.4%)	4 (0.8%)	0.68
CKD	28 (5.4%)	30(5.8%)	0.96
**Clinical parameters**			
SBP, mmHg, mean ±SD	144.9±28.4	144.5±26.3	0.81
DBP, mmHg, mean ± SD	80.8±16.0	81.1±15.0	0.73
HR, bpm, mean ± SD	85.4±20.2	85.3±20.2	0.91
LVEF, %	53.5±8.5	53.9±8.2	0.50
**Medications prior to admission**			
Aspirin	421 (82.4%)	318 (62.2%)	<0.001
ACE-inhibitors and/or ARBs	342 (66.9%)	369 (72.2%)	0.06
Statins	423 (82.8%)	346 (67.7%)	<0.001
Clopidogrel or prasugrel	152 (29.7%)	73 (14.3%)	<0.001

Data are shown as number (percentage) or mean ± standard deviation. TIAs=transient ischemic attacks, PAD= peripheral artery disease, CKD = chronic kidney disease, SBP= systolic blood pressure, DBP= diastolic blood pressure, LVEF= Left ventricular ejection fraction, HR= heart rate, ARBs= angiotensin receptor blockers.

Supplementary Table 2: Baseline characteristics of propensity-score matched patients discharged alive from ACS in the GULF-COAST, with a LVEF >= 40%, according to beta-blockers on discharge.

**Variable**	**Beta-blockers on discharge**<br><br>**N= 291**	**No beta-blockers on discharge**<br><br>**N= 291**	**p**
**Demographics**			
Age (years)	60.6±12.1	60.8±14	0.70
Gender (Male)	152 (52.2)	151 (51.9%)	0.59
BMI (kg/m2)	30.6±6.4	30.8±9.6	0.23
Smoking (current)	75 (25.7%)	73 (25.1%)	0.69
**Past medical history**			
Dyslipidemia	161 (55.3%)	160 (55.0%)	0.49
Hypertension	190 (65.3%)	193 (66.3%)	0.56
Diabetes	150 (51.5%)	144 (49.5%)	0.47
Myocardial infarction	55 (18.9%)	50 (17.2%)	0.48
Stroke/TIAs	20 (6.8%)	19 (6.5%)	0.25
PAD	10 (3.4%)	9 (3.1%)	0.27
Cancer	2 (0.7%)	3 (1.0%)	0.25
Venous thrombo-embolism	2 (0.7%)	1 (0.3%)	0.65
CKD	17 (5,7%)	20 (6.8%)	0.64
**Clinical parameters**			
SBP, mmHg, mean ±SD	144.9±28.4	139.2±27.5	0.18
DBP, mmHg, mean ± SD	80.8±16.0	77.5±15.3	0.37
HR, bpm, mean ± SD	82.2±22.4	81.8±22.1	0.21
LVEF, %	53.2±8.3	52.8±8.4	0.25
**Medications at discharge**			
Aspirin	284 (97.6%)	269 (92.4%)	0.01
ACE-inhibitors and/or ARBs	215 (73.9%)	199 (68.4%)	0.04
Statins	280 (96.2%)	268 (92.1%)	0.02
Clopidogrel or prasugrel	200 (68.7%)	184 (63.2%)	0.03
**Discharge diagnosis**			
STEMI	52 (19.7%)	45 (17.2%)	0.11

NSTEMI	130 (50.1%)	136 (52.1%)	0.64
Unstable Angina	79 (30.2%)	80 (30.7%)	0.63
**Treatment**			
PCI *	88 (30.2%)	65(22.3%)	0.03
Thrombolysis	40 (13.7%)	30 (10.3)	0.20
CABG	5 (1.7%)	5 (1.7%)	0.47
**Severity of lesions****			
1 artery	39 (36.8%)	35 (41.6%)	0.85
2 arteries	27 (25.5%)	21 (25%)	
3 arteries	30 (28.3%)	20 (23.8%)	
4 arteries	10 (9.4%)	8 (9.5%)	

Data are shown as number (percentage) or mean ± standard deviation. TIAs=transient ischemic attacks, PAD= peripheral artery disease, SBP= systolic blood pressure, DBP= diastolic blood pressure, HR= heart rate, LVEF= Left ventricular ejection fraction, HR= heart rate, ARBs= angiotensin receptor blockers, ARBs= angiotensin receptor blockers, STEMI-ST elevation myocardial infarction, NTSEMI= Non- ST-elevation myocardial infarction. PCI= percutaneous coronary intervention, CABG= coronary artery bypass graft. *including primary PCI. ** among patients who underwent PCI.
